# CD99 antibody disrupts T-cell acute lymphoblastic leukemia adhesion to meningeal cells and attenuates chemoresistance

**DOI:** 10.1038/s41598-021-03929-x

**Published:** 2021-12-21

**Authors:** Maryam Ebadi, Leslie M. Jonart, Jason Ostergaard, Peter M. Gordon

**Affiliations:** 1 Division of Pediatric Hematology and Oncology, Department of Pediatrics, University of Minnesota, 420 Delaware St SE, MMC 366, Minneapolis, MN 55455 USA; 2 Masonic Cancer Center, University of Minnesota, Minneapolis, MN USA

**Keywords:** Cancer microenvironment, Cancer therapy, Haematological cancer

## Abstract

Central nervous system (CNS) relapse is a significant cause of treatment failure among patients with acute lymphoblastic leukemia. In prior work we found that the meninges, the thin layer of tissue that covers the brain and spinal cord, harbor leukemia cells in the CNS. Importantly, direct interactions between leukemia and meningeal cells enabled leukemia chemoresistance. Herein, we show that an antibody targeting CD99, a transmembrane protein expressed on meningeal cells and many leukemia cells, disrupts adhesion between leukemia and meningeal cells and restores sensitivity of the leukemia cells to chemotherapy. This work identifies a mechanism regulating critical intercellular interactions within the CNS leukemia niche and may lead to novel therapeutic approaches for overcoming niche-mediated chemoresistance.

## Introduction

Current treatment modalities for acute lymphoblastic leukemia (ALL) yield ~ 90% cure rate in children and ~ 45% in adults^1,2^. However, relapse in the central nervous system (CNS) remains a significant cause of treatment failure despite intensive, and often toxic, CNS directed therapies^3,4^. We previously showed that direct interactions between leukemia and meningeal cells in the CNS enhance leukemia chemotherapy resistance through effects on leukemia apoptosis balance and cell cycle^5,6^. We then showed that Me6TREN (Tris[2-(dimethylamino) ethyl]amine), a small molecule drug initially identified as a hematopoietic stem cell (HSC) mobilizing compound^7,8^, disrupts leukemia-meningeal cell adhesion and significantly overcomes leukemia chemotherapy resistance both in vitro and in vivo. While the mechanism by which Me6TREN regulates cellular adhesion is likely multifactorial, gene expression profiling identified the transmembrane glycoprotein CD99 as being altered in meningeal cells treated with Me6TREN.

CD99 is cell surface protein expressed on multiple different cell types, including lymphocytes, that is involved in a range of cellular processes including cellular adhesion and migration, apoptosis and cell survival, T-cell differentiation, and cancer biology^9,10^. Moreover, CD99 is often expressed on myeloid and lymphoid leukemia cells and can both aid in leukemia diagnosis and correlate with prognosis^11–14^. In addition, a CD99 monoclonal antibody (clone H036 1.1) has been shown to be directly toxic to acute myeloid leukemia (AML) and myelodysplastic syndrome (MDS) cells and may be a novel therapeutic approach for the treatment of these myeloid malignancies^15^.

Herein, we explored the role of CD99 in leukemia-meningeal interactions. We showed that CD99 is expressed on meningeal cells and that CD99 ligation with a monoclonal antibody disrupts adhesion between leukemia and meningeal cells and restores sensitivity of the leukemia cells to chemotherapy. Moreover, the ability of the CD99 antibody to disrupt adhesion was dependent upon matrix metalloprotease activity. This work provides insights into the role CD99 plays in the CNS leukemia niche and supports directly targeting CD99, or modulating its downstream pathways, as potential approaches for overcoming meningeal-mediated leukemia chemoresistance.

## Results and discussion

**Figure 1:**
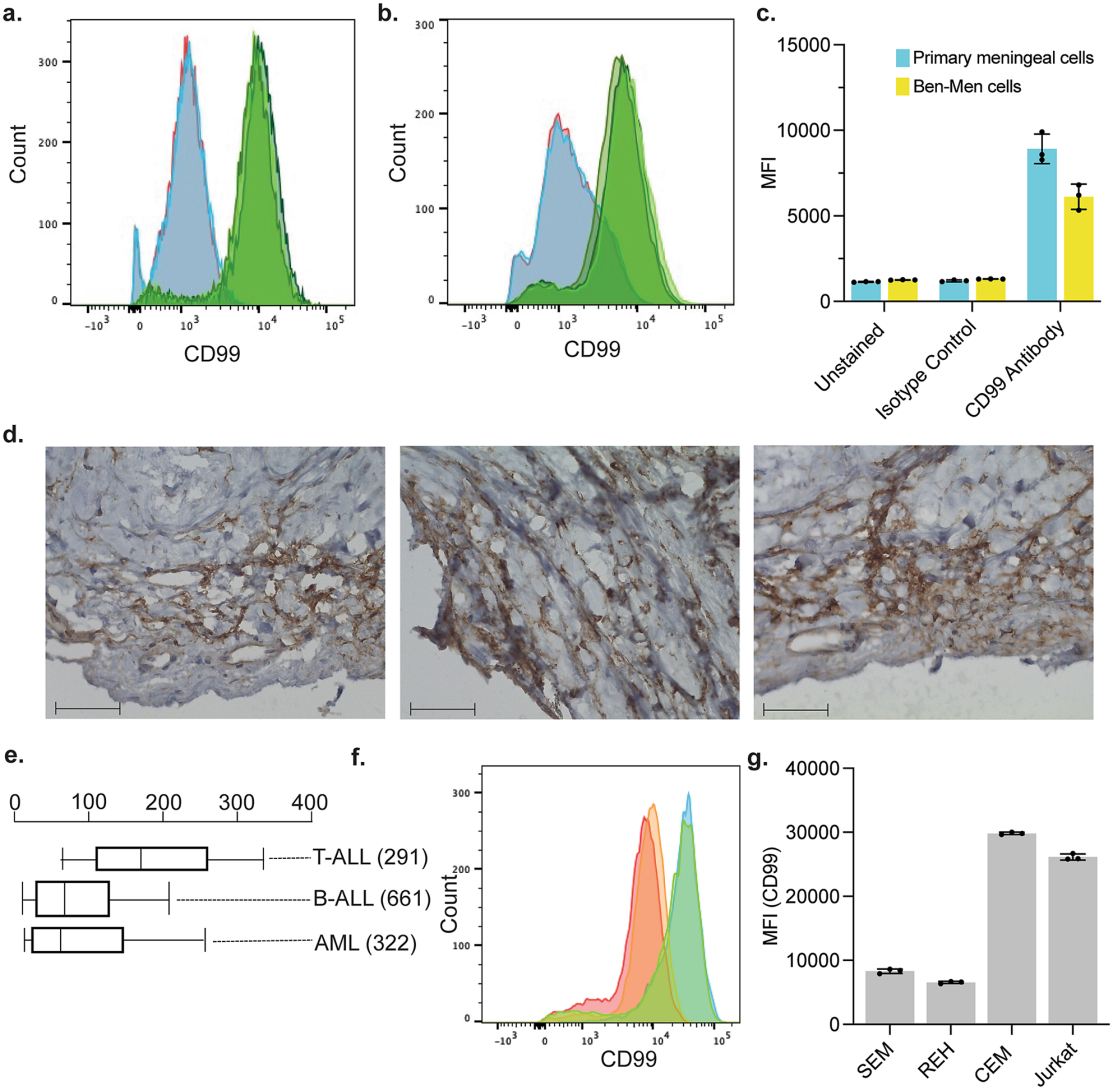
Human meningeal and leukemia cells express CD99. (**a**–**c**) Primary human meningeal cells (**a**; n = 3) and the meningeal cell line Ben-Men (**b**) were unstained (red), stained with either an isotype control antibody (blue; IgG2a kappa-FITC) or a CD99 antibody (green; CD99-FITC), assessed by flow cytometry, and median fluorescent intensity (MFI) calculated (**c**). (**d**) Immunohistochemistry for human CD99 was performed on three formalin-fixed paraffin-embedded tissue sections of normal human meninges. CD99 positive cells stain brown. Magnification 40× and scale bar 50 μm. € CD99 mRNA expression in primary pediatric hematologic cancer samples was determined using RNA-seq data from the St. Jude Cloud database (https://www.stjude.cloud). The numbers in parentheses represent the number of samples in each group. (**f**, **g**) REH (B-ALL, red), SEM (B-ALL, orange), CEM (T-ALL, green) and Jurkat (T-ALL, blue) were stained with a CD99 antibody, assessed by flow cytometry (**f**), and median fluorescent intensity (MFI) calculated (**g**). For all graphs, data are the mean ± SD, circles represent individual data points, and the results are representative of three independent experiments.

We previously used RNA-seq to identify alterations in CD99 mRNA levels in meningeal cells treated with Me6TREN^5^. Accordingly, we first confirmed the expression of CD99 protein on meningeal cells. Primary human meningeal cells and a meningeal cell line (Ben-Men^16^) grown ex vivo were stained with a human CD99 antibody (eBioScience, clone: 3B2/TAB) and assessed by flow cytometry. Both the primary meningeal cells and meningeal cell line highly expressed CD99 (Fig. 1a–c). To extend this result in vivo, we next used immunohistochemistry to examine CD99 expression on human meningeal tissue sections. As shown in Fig. 1d, the meningeal tissue sections exhibited CD99-positive cells and confirmed the tissue culture results. Finally, we confirmed prior work demonstrating CD99 expression in leukemia cells. We used the St. Jude PeCan Data Portal^17^ to assess CD99 mRNA expression in > 1000 pediatric hematologic malignancy cases and found CD99 is more highly expressed in pediatric T-cell leukemia than either B-cell leukemia or AML (Fig. 1e). In agreement, we found that T-ALL cell lines (CEM and Jurkat) expressed higher levels of CD99 protein than B-ALL cell lines (SEM and REH) (Fig. 1f,g). Based on these data, we elected to utilize T-ALL cell lines (CEM and Jurkat) and primary T-ALL samples for subsequent experiments.

**Figure 2:**
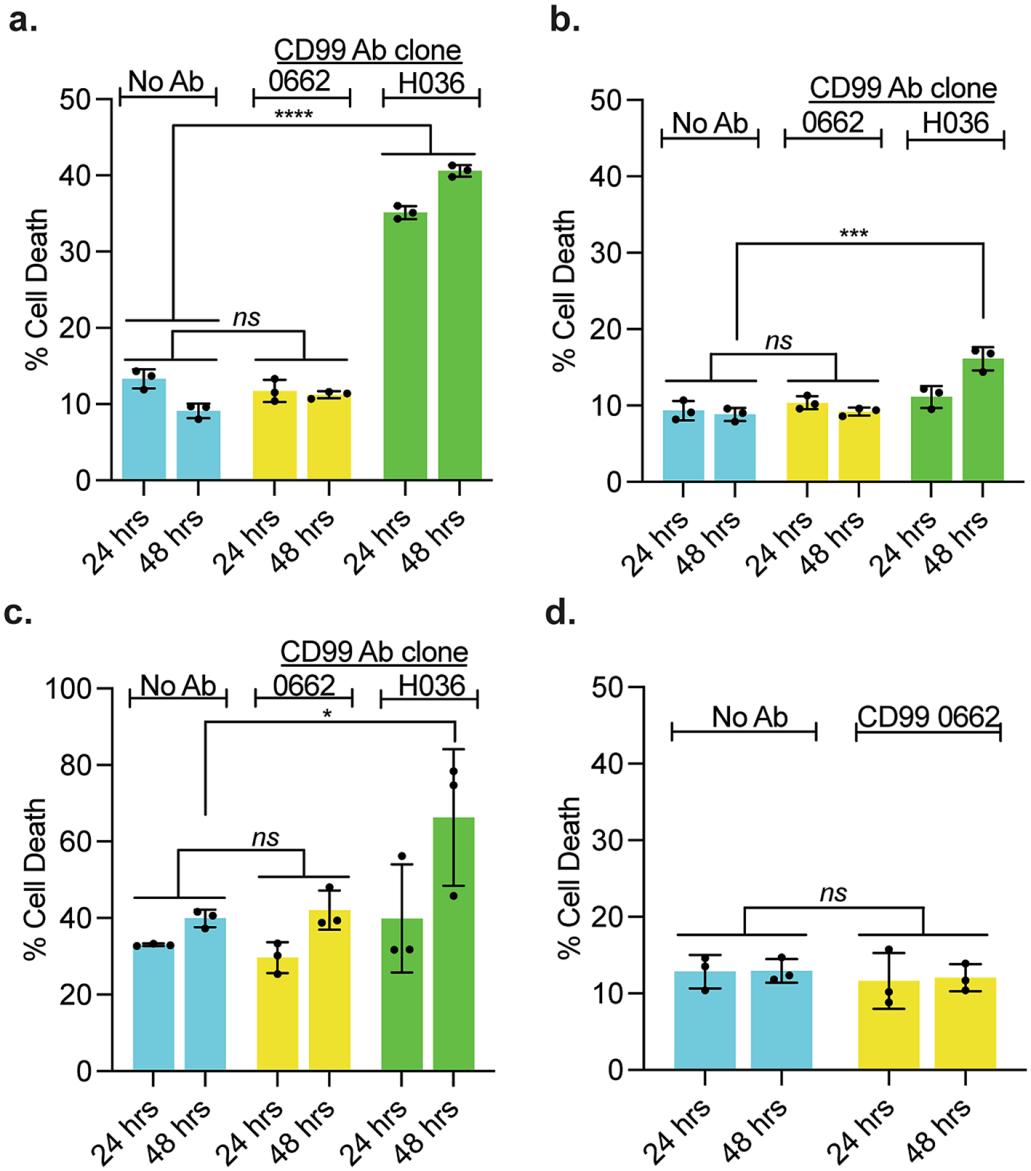
CD99 antibody clone 0662 is non-toxic to leukemia and meningeal cells. (**a**–**d**) Jurkat (**a**), CEM (**b**), T-ALL PDX (**c**), and human meningeal (**d**) cells were incubated with either CD99 antibody clone 0662 (5 μg/mL) or CD99 antibody clone H036 1.1 (5 μg/mL) and viability measured at 24 and 48 h with annexin-V/7AAD staining and flow cytometry. For all graphs, data are the mean ± SD, circles represent individual data points, and the results are representative of three independent experiments. *P*: ***, < 0.001, ****, < 0.0001 by ANOVA.

We next sought to test the ability of a CD99 antibody to modulate the adhesion of leukemia and meningeal cells. However, prior to these experiments we examined the effect of CD99 antibodies (clones 0662 and H036 1.1) on leukemia cell viability as a CD99 monoclonal antibody (clone H036 1.1) has previously been shown to cause cell death in AML and MDS cells both in vitro and in xenografts^15^. CD99 antibody clone H036 1.1 caused modest ALL cell death (Fig. 2a–c). In contrast, CD99 antibody clone 0662 was not toxic to Jurkat and CEM cell lines or primary T-ALL cells at either 24 or 48 h (Fig. 2a–c). Finally, CD99 antibody clone 0662 had no effect on meningeal cell viability (Fig. 2d). Based on these results, we selected the CD99 antibody clone 0662 for further testing in leukemia-meningeal adhesion assays.

**Figure 3:**
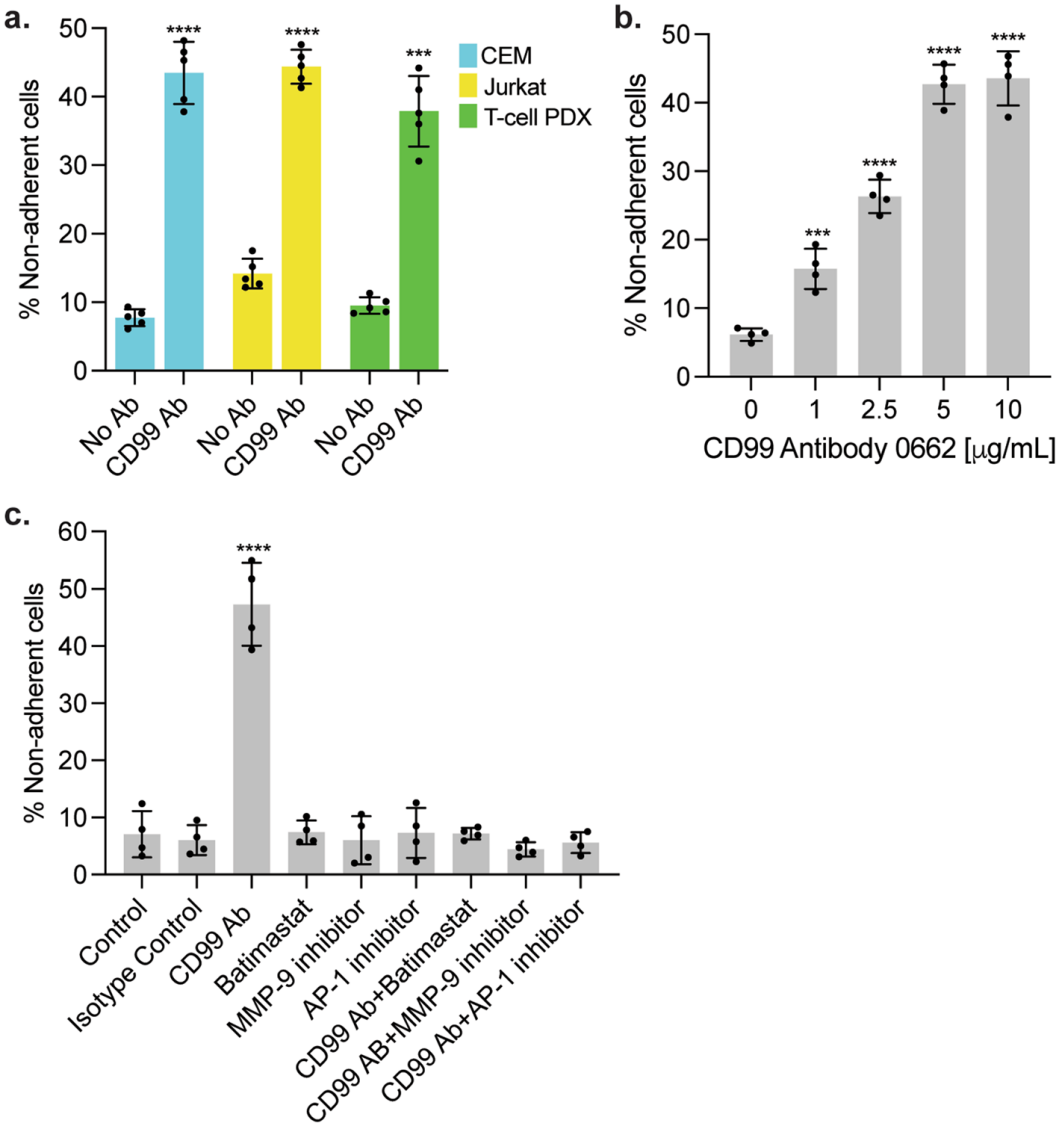
CD99 antibody disrupts leukemia-meningeal cell adhesion. (**a**) CEM, Jurkat and T-cell PDX leukemia cells were co-cultured with human meningeal cells in the presence or absence of CD99 antibody clone 0662 5 μg/mL. After 24 h, non-adherent leukemia cells were removed and quantitated using count beads and flow cytometry. (**b**) Jurkat leukemia cells were co-cultured with human meningeal cells over a range of CD99 antibody clone 0662 concentrations. After 24 h, non-adherent leukemia cells were removed and quantitated using count beads and flow cytometry. (**c**) Jurkat leukemia cells were co-cultured with primary meningeal cells in the presence or absence of isotype control antibody (mouse IgG3, kappa monoclonal) 5 μg/mL or CD99 antibody clone 0662 5 μg/mL ± batimastat 500 nM, a MMP-9 inhibitor 1 μM, or an AP-1 inhibitor 500 nM. After 24 h, non-adherent leukemia cells were removed and quantitated using count beads and flow cytometry. For all graphs, data are the mean ± SD, circles represent individual data points, the results are representative of three independent experiments. *P*: **, < 0.01, ***, < 0.001, ****, < 0.0001 by t-test or ANOVA.

To test the effect of CD99 antibody on leukemia-meningeal cell adhesion, Jurkat, CEM and primary T-ALL cells were cultured on ~ 80% confluent primary, human meningeal cells in the presence or absence of CD99 antibody clone 0662. After 24 h, non-adherent leukemia cells in suspension were collected and counted using count beads and flow cytometry. We observed significantly more non-adherent leukemia cells in the presence of CD99 antibody suggesting that CD99 ligation by CD99 antibody clone 0662 disrupted the adhesion of leukemia and meningeal cells (Fig. 3a). We also tested the effect on adhesion of separately pre-treating both the meningeal and leukemia cells with CD99 antibody. We found that CD99 ligation by antibody on either cell type was sufficient to disrupt subsequent adhesion (Supplementary Fig. 1). We next performed a CD99 antibody dose–response titration and found the number of non-adherent leukemia cells was proportional to the concentration of CD99 antibody (Fig. 3b).

We also explored the mechanism by which antibody ligation of CD99 on leukemia and meningeal cells may disrupt leukemia-meningeal cell adhesion. While CD99 participates in homo-, and likely hetero-typic, intercellular interactions the ligation of CD99 also activates multiple different intracellular downstream signaling pathways that also potentially regulate cellular adhesion and migration^9,18–23^. A long-term goal of our research is to use genomic and proteomic approaches to define the effects of CD99 ligation on signaling pathways in leukemia and meningeal cells. However, while these studies are underway, we also investigated the matrix metalloprotease (MMP) pathway for several reasons. First, in prior work we showed that that MMP activation contributed to the disruption of leukemia-meningeal adhesion^5^. Second, the MMP pathway was recently shown to regulate ALL progression and invasion of multiple tissues including the meninges^24^. Third, antibody ligation of CD99 upregulated matrix metalloprotease 9 (MMP-9) expression, via binding of AP-1 transcription factors to the MMP-9 gene promoter, in breast cancer cell lines and increased cell motility^25^. Accordingly, we next tested whether MMP activity is required for the ability of the CD99 antibody to disrupt leukemia-meningeal cell adhesion. We performed the adhesion assay in the presence of the broad spectrum MMP inhibitor Batimastat, a more specific MMP-9 inhibitor, or AP-1 inhibitor T-5224. As shown in Fig. 3c, all three inhibitors significantly inhibited the ability of CD99 antibody to disrupt leukemia adhesion to meningeal cells. This effect on adhesion was not secondary to any toxicity of the drugs, alone or in combination with CD99 antibody, against leukemia or meningeal cells (Supplementary Fig. 2a,b). These data support a role of the MMP pathway in disrupting adhesion, perhaps through remodeling of the extracellular matrix.

**Figure 4:**
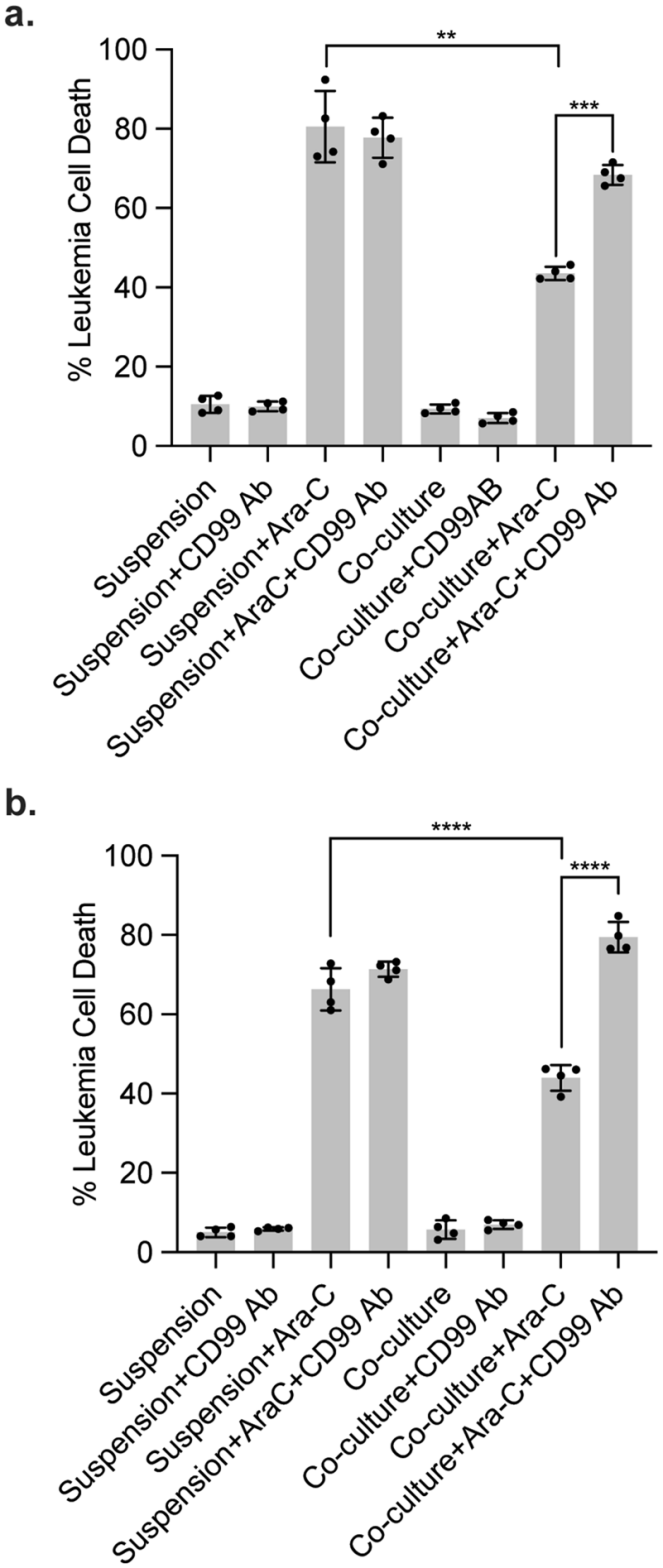
CD99 antibody meningeal-mediated leukemia chemoresistance. CEM (**a**) and Jurkat (**b**) leukemia cells in suspension or co-cultured with primary meningeal cells were treated with CD99 antibody clone 0662 5 μg/mL ± cytarabine 1 μM and viability assessed with annexin-V/7AAD staining and flow cytometry. Data are the mean ± SD, circles represent individual data points, and *P*: ***, < 0.001, ****, < 0.0001 by ANOVA. The results are representative of two independent experiments.

We previously showed that direct leukemia-meningeal cell interactions enabled leukemia chemoresistance in the CNS^5^. However, this effect was reversible and leukemia chemoresistance overcome by detaching the leukemia cells from the meninges. Accordingly, we hypothesized that CD99 antibody clone 0662 should overcome meningeal-mediated leukemia chemoresistance by disrupting leukemia-meningeal adhesion. To test this, we performed the adhesion assay in the presence of CD99 antibody and cytarabine. Jurkat and CEM leukemia viability was then assessed using annexin-V/7AAD staining and flow cytometry. In agreement with prior results, leukemia cells co-cultured with primary meningeal cells were significantly more resistant to cytarabine than the same leukemia cells in suspension (Fig. 4a,b). However, the addition of CD99 antibody significantly reversed meningeal-mediated leukemia chemoresistance and increased leukemia cell death. This result, and our prior work^5,6^, suggest that any therapeutic agent (drug, antibody) that disrupts leukemia-meningeal adhesion may enhance the efficacy of chemotherapy in the treatment of CNS leukemia.

In summary, these data show that the meninges express CD99 and that a CD99 antibody disrupts the adhesion of leukemia and meningeal cells and, in doing so, increases the efficacy of chemotherapy. Disruption of the CNS leukemia niche, with a CD99 antibody or by other mechanisms, represents a novel therapeutic approach for enhancing the ability of chemotherapy to eradicate CNS leukemia and prevent relapse. While the CD99 antibody was efficacious in overcoming meningeal-mediated leukemia chemoresistance ex vivo, the clinical utility of a systemically delivered CD99 antibody would likely be limited due to the typically poor penetration of antibodies across the blood–brain/blood–cerebrospinal fluid barriers and into the CNS^26^. However, this limitation could be addressed by engineering the CD99 antibody to more efficaciously cross these CNS barriers via receptor mediated transport^27,28^. Alternatively, ALL patients receive intrathecal chemotherapy throughout the duration of therapy to bypass the blood brain barrier. Accordingly, a CD99 antibody could be delivered intrathecally at the same time as chemotherapy administration. Finally, our further efforts to define the signaling pathways downstream of CD99 ligation may identify alternative approaches for activating MMPs and disrupting leukemia-meningeal adhesion that are more readily targetable with a small molecule drug that penetrates the CNS.

## Methods

### Cells, tissue culture, reagents

CEM, Jurkat, SEM, and REH leukemia cell lines were obtained from American Type Culture Collection (ATCC) or DSMZ and cultured in RPMI media (Sigma-Aldrich) supplemented with Fetal Bovine Serum (FBS; Seradigm) 10% and Penicillin–Streptomycin (Sigma-Aldrich). The human meningeal cell line Ben-Men was obtained from DSMZ and cultured in DMEM media (Sigma-Aldrich) supplemented with FBS 10% and Penicillin-Streptomycin^16^. Primary human meningeal cells were obtained from ScienCell and cultured in meningeal media (ScienCell) plus FBS 2%, growth supplement, and Penicillin-Streptomycin^5^. Meningeal cells were from multiple different donor specimens and were used between passages 3–5. Primary T-ALL cells (sample CBAT-44179-V1) were obtained from the Public Repository of Xenografts (PRoXe). Mouse IgG2a kappa isotype control-FITC and anti-human CD99-FITC antibodies were from eBioscience.

### Cell adhesion assay

Leukemia cells were labelled with cell trace violet dye (ThermoFisher Scientific) and cultured on ~ 80% confluent meningeal cells. Anti-human CD99 monoclonal antibody clone 0662 (MABF927, EMD Millipore) or isotype control (mouse IgG3, kappa monoclonal; Abcam ab18394) were added at the same time as co-culture. For some experiments, MMP-9 inhibitor I 1 μM (EMD Millipore CAS 1177749-58-4), AP-1 inhibitor T-5224 500 nM (Fisher Scientific) or Batimastat 500 nM (BioVision) were added as well. After 24 h, the supernatant was collected and count beads were used with flow cytometry to determine the number of non-adherent, viable leukemia cells. Labeling of the leukemia cells with cell trace violet facilitated gating out any non-adherent meningeal cells. The ratio of leukemia cells in each condition to cells plated were used to calculate the percentage of non-adherent leukemia cells. For experiments testing the effect of CD99 antibody pre-treatment, Jurkat and human meningeal cells were separately incubated ± CD99 antibody clone 0662 (5 μg/mL) for 24 h, washed to remove excess antibody, combined in a co-culture adhesion assay, and adhesion measured after an additional 4 h.

### Immunohistochemistry

Unstained human meningeal sections (5 µm; Amsbio, T2234043) were de-paraffinized and rehydrate. For antigen retrieval, slides were incubated in pH 6.0 buffer (Reveal Decloaking reagent, Biocare Medical) at 95–98 °C followed for 30 min followed by a 20 min cool down period. Subsequent steps utilized an immunohistochemical staining platform (Intellipath, Biocare). Slides were immersed in a 3% hydrogen peroxide solution (Peroxidazed, Biocare) for 10 min, rinsed with TBST and blocked using a serum-free solution (Background Sniper, Biocare Medical, Concord, CA) for 10 min. Blocking solution was removed and slides were incubated with rabbit monoclonal anti-CD99 antibody (Clone EPR3097Y; Cell Marque) diluted in 10% blocking solution/90% TBST for 60 min at room temperature. Slides were then washed with TBST and detection performed with the Novocastra Novolink Polymer Kit (Leica Microsystems Inc.) according to the manufacturer’s specifications. Slides were then rinsed with TBST, detection performed with diaminobenzidine (DAB; Biolegend) and counterstained with CAT Hematoxylin (Biocare). Finally, slides were dehydrated and coverslipped.

### Cell viability and chemoresistance assays

Leukemia or meningeal cells were incubated with anti-CD99 monoclonal antibody clone 0662 (MABF927, EMD Millipore) or clone H036 1.1 (ab23898, Abcam) and viability assessed at 24 and 48 h with annexin-v/7AAD staining and flow cytometry. For chemoresistance assays, leukemia cells were cultured with ~ 70% confluent meningeal cells in the presence or absence of anti-CD99 monoclonal antibody clone 0662 (MABF927, EMD Millipore) 5 μg/mL. After 24 h of co-culture, Ara-C 1 μM was added and the cells were re-treated with the CD99 antibody. Leukemia cells were harvested after an additional 24 h and apoptosis was measured by annexin-v/7AAD staining and flow cytometry.

### Statistical analysis

Statistical analyses were conducted using GraphPad Prism 9 software (GraphPad Software, La Jolla, CA). Results are shown as the mean plus or minus the SD. The Student’s *t*-test or ANOVA were used for statistical comparisons between groups. *P*-values < 0.05 were considered statistically significant.

## Supplementary Information

Supplementary Information.
