# Supplementary material for: CD99 antibody disrupts T-cell acute lymphoblastic leukemia adhesion to meningeal cells and attenuates chemoresistance
Source: Sci Rep. 2021 Dec 21;11:24374. doi: 10.1038/s41598-021-03929-x (PMC8692434; doi:10.1038/s41598-021-03929-x)
Supplement: Supplementary file 1 — Supplementary Information. [file 41598_2021_3929_MOESM1_ESM.pdf]

CD99 antibody disrupts T-cell acute lymphoblastic leukemia adhesion to meningeal cells and attenuates chemoresistance; Maryam Ebadi, Leslie M. Jonart, Jason Ostergaard, and Peter M. Gordon

Supplementary Figure S1

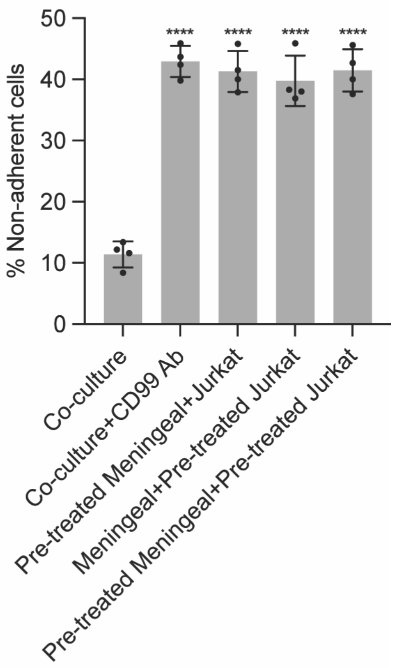

Supplementary Figure S1: ***CD99 antibody pre-treatment of either leukemia or meningeal cells can disrupt subsequent leukemia-meningeal adhesion in co-culture*.** Jurkat leukemia and human meningeal cells were separately incubated +/- CD99 antibody clone 0662 (5 μg/mL) for 24 hours, washed to remove excess antibody, and then combined in a co-culture adhesion assay. Adhesion was measured at 4 hours. Data are the mean +/- SD of three technical replicates, circles represent individual data points, and the results are representative of three independent experiments. *P*: ****, <0.0001 by ANOVA.

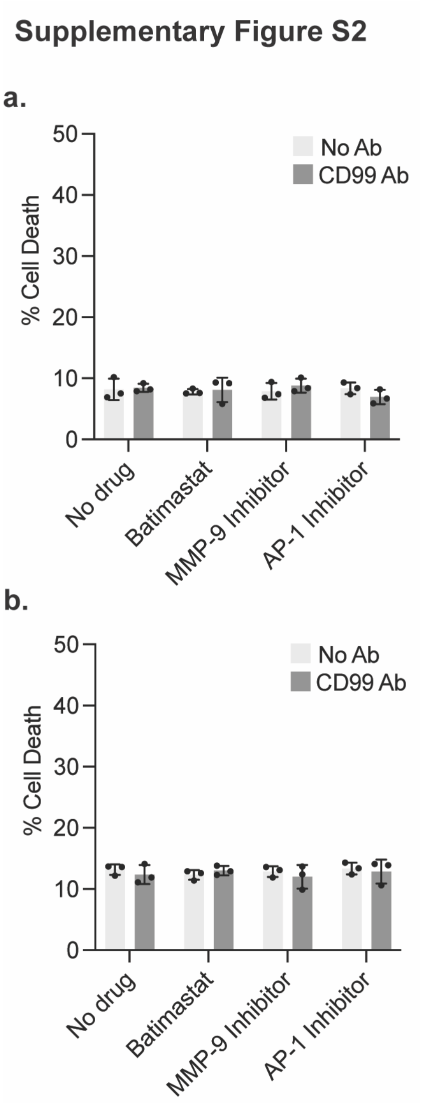

Supplementary Figure S2: The combination of *CD99 antibody with AP-1 or MMP inhibitors is non-toxic to leukemia and meningeal cells.* A-B. Jurkat (**A**) and human meningeal (**B**) cells were incubated either with CD99 antibody clone 0662 (5 μg/mL) or without antibody in the presence or absence of batimastat 500 nM, a MMP-9 inhibitor 1 μM, or an AP-1 inhibitor 500 nM. Viability was measured at 48 hours with annexin-V/7AAD staining and flow cytometry. For both graphs, data are the mean +/- SD of three technical replicates, circles represent individual data points, and the results are representative of three independent experiments. There are no significant differences between conditions by ANOVA.
